# Does kidney function in experimental porcine sepsis fulfill the RIFLE criteria for acute kidney injury? A systematic review

**DOI:** 10.3389/fphys.2026.1810924

**Published:** 2026-08-19

**Authors:** Kjellbjørn Jakobsen, Viktoria Instanes Markussen, Hendrik Joachim Backmann, Eirik Reierth, Anders Benjamin Kildal, Lars Marius Ytrebø

**Affiliations:** 1 Anesthesia and Critical Care Research Group, Institute of Clinical Medicine, UiT - The Arctic University of Norway, Tromsø, Norway; 2 Department of Anesthesiology and Intensive Care, Division of Surgery, University Hospital of North Norway, Tromsø, Norway; 3 University Library, Science and Health Library, UiT - The Arctic University of Norway, Tromsø, Norway

**Keywords:** acute kidney injury, creatinine, diuresis, glomerular filtration rate, sepsis, swine

## Abstract

**Introduction:**

Sepsis is a leading cause of acute kidney injury (AKI) in patients treated in intensive care units. Due to close resemblance between porcine and human anatomy and physiology, porcine models are increasingly utilized in investigations of pathophysiological mechanisms behind sepsis associated AKI.

**Objective:**

The aim of this review was to assess the renal function in untreated porcine models of sepsis using the Risk, Injury, Failure, Loss, End-stage kidney disease (RIFLE) criteria at the group level.

**Design:**

The review was pre-registered in the Open Science Framework, DOI 10.17605/OSF.IO/FC29M. A systematic literature search was performed to identify porcine sepsis models which fulfilled the RIFLE criteria in the absence of active treatment. Three databases (Medline, Embase, and Web of Science) were systematically searched from 1946 until April 29^th^, 2025. *In vivo* studies aiming to induce sepsis or sepsis like conditions were included if the RIFLE criteria could be assessed at the group level in at least one study group. A meta-analysis was performed to study the relation between sepsis induction method and the likelihood of fulfilling the RIFLE criteria.

**Results:**

1270 records were retrieved via databases and registers. Additional 309 records were identified from citation searching. 64 studies were included in the systematic review. 30 of 64 (47%) studies fulfilled one of the RIFLE criteria. 16, 9 and 5 studies fulfilled the RIFLE Risk, Injury and Failure criteria, respectively. 28 out of 64 studies (44%) reported renal dysfunction, but only two studies defined AKI using a diagnostic criterion. Exogenous markers of glomerular filtration rate (GFR) were applied in three studies. Fulfilment of the RIFLE criteria was associated with GFR being reported (*p* = 0.007), while the meta-analysis found no difference in the rate of AKI between different sepsis induction methods (Q_between = 2.49, *p* = 0.48).

**Conclusions:**

AKI at the group level was identified in 47% of the included studies. Exploratory meta-analyses did not identify any statistically robust differences in RIFLE fulfilment between sepsis induction methods, but methodological heterogeneity significantly limited the interpretability of the meta-analysis. Measurement of GFR was associated with a higher likelihood of RIFLE fulfilment, while the use of precise markers appeared limited.

## Introduction

Sepsis leads to life-threatening organ dysfunction caused by a dysregulated host response to infection (Rhodes et al., 2017). Sepsis is a common feature in critically ill patients and sepsis associated acute kidney injury (SA-AKI) develops in up to 70% of the patients (Ostermann et al., 2025). Despite considerable efforts over several decades, pathogenesis of acute kidney injury (AKI) remains elusive, and this lack of understanding represents a major barrier for development of effective therapies. Experimental studies are pivotal, and pigs are of particular interest in this context due to the resemblance between pig and human kidney anatomy and physiology. Porcine models have thus been considered ideal for studies of AKI and SA-AKI (Huang et al., 2021). However, as not all septic patients are afflicted by AKI, it is reasonable to assume that the same is true for pigs as well. Thus, a formal definition of AKI could ease the assessment of whether a porcine model of sepsis is relevant for the study of SA-AKI. Similarly, it may facilitate grading of the severity of renal insufficiency within and across various models. To the best of our knowledge, no single definition of AKI has seen broad use in porcine models of sepsis.

Similarly, the long-lasting lack of a consensus definition of AKI in humans made the design of both experimental and clinical studies challenging and complicated interpretation of data. The first international interdisciplinary consensus definition of acute renal failure (ARF) in humans was established in 2004. The Second International Consensus Conference of the Acute Dialysis Quality Initiative (ADQI) Group performed an extensive literature search, and through its results and by consensus agreement they developed a classification system (Bellomo et al., 2004). This new system was named RIFLE, which is an acronym for specific steps in the new grading system (Risk, Injury, Failure). The last two letters refer to two outcome parameters (Loss and End-stage kidney disease) (Bellomo et al., 2004). Grading of ARF was based on two criteria, dependent upon three parameters: relative increase in serum creatinine or decrease in GFR (collectively: GFR criteria), and urine output (urine output criteria). Moreover, in 2007 the Acute Kidney Injury Network (AKIN) endorsed the RIFLE criteria with notably a modification to also include small acute increases in serum creatinine (≥0.3 mg/dL or 26.5 μmol/l), due to new evidence suggesting that this was associated with increased mortality (Mehta et al., 2007). Additionally, the GFR parameter was removed. The AKIN criteria introduced the term AKI into the new classification system with a stepwise approach through stages 1-3, with increasing severity. In essence, AKI stage 1 corresponded with RIFLE Risk, while stages 2 and 3 corresponded with Injury and Failure, respectively. This AKI definition was largely unchanged in the Kidney Disease: Improving Global Outcomes (KDIGO) guideline for AKI in 2012 (Kellum et al., 2012), which is the current broadly accepted AKI definition in humans. Thus, the current AKI definition remains true to the original RIFLE thresholds (e.g. the percentwise increase in creatinine, the duration and degree of oliguria) and the structure of staging severity. **Table 1** provides details of these three classification systems for AKI.

**Table 1:** Definitions and staging criteria for acute kidney injury.

RIFLE	S-creatinine and GFR criteria	Urine output criteria
Risk	Increase in s-creatinine to 1.5 times baseline OR 25% decrease in GFR	<0.5 mL/kg/h for ≥6 hours
Injury	Increase in s-creatinine to 2 times baseline OR 50% decrease in GFR	<0.5 mL/kg/h for ≥12 hours
Failure	Increase in s-creatinine to 3 times baseline OR 75% decrease in GFR OR s-creatinine 354 μmol/L (>4 mg/dL) in the setting of an acute increase of at least 44 μmol/L (0.5 mg/dL)	<0.3 mL/kg/h for ≥24 hours OR anuria for ≥12 hours
Loss	Persistent acute renal failure = complete loss of kidney function > 4 weeks	
End Stage Kidney Disease	End stage renal disease (on dialysis for >3 months)	
AKIN	S-creatinine criteria	Urine output criteria
Stage 1	≥26.5 μmol/L (0.3 mg/dL) increase in serum creatinine OR increase in s-creatinine to 1.5 - 2.0 times baseline.	<0.5 mL/kg/h for ≥6 hours
Stage 2	Increase in s-creatinine to 2–3 times baseline	<0.5 mL/kg/h for ≥12 hours
Stage 3	Increase in s-creatinine to >3 times baseline, OR s-creatinine 354 μmol/L (>4 mg/dL) in the setting of an acute increase of at least 44 μmol/L (0.5 mg/dL)) OR on renal replacement therapy	<0.3 mL/kg/h for ≥24 hours OR anuria for ≥12 hours
KDIGO	S-creatinine criteria	Urine output criteria
Stage 1	Increase in serum creatinine to 1.5 - 1.9 times baseline OR ≥26.5 μmol/L (0.3 mg/dL)	<0.5 mL/kg/h for 6–12 hours
Stage 2	Increase in serum creatinine to 2.0 - 2.9 times baseline	<0.5 mL/kg/h for ≥12 hours
Stage 3	Increase in s-creatinine to 3.0 times baseline OR increase in s-creatinine to 353.6 μmol/L (>4 mg/dL) OR initiation of renal replacement therapy	<0.3 mL/kg/h for ≥24 hours OR anuria for ≥12 hours

The table summarizes the staging criteria for acute kidney injury according to the RIFLE, AKIN, and KDIGO classification systems.

AKIN, Acute Kidney Injury Network; KDIGO, Kidney Disease: Improving Global Outcomes; RIFLE, Risk, Injury, Failure, Loss, End-stage kidney disease; GFR, glomerular filtration rate; s-creatinine, serum creatinine.

The human staging systems were invented out of necessity for improving the quality of clinical research and treatment protocols. Indeed, large studies covering over 500 000 subjects have later validated RIFLE and/or AKIN for diagnosis and staging of AKI in humans (Kellum et al., 2012). The risk and need for renal replacement therapy correlates with increased stage, demonstrating the usefulness of the staging systems (Kellum et al., 2012). The lack of staging systems for use in experimental research in porcine models represent a similar obstacle. Due to the similarities between human and porcine renal anatomy and physiology, criteria developed for the diagnosing and staging of AKI in humans may also be relevant in porcine models.

Among the available human classification systems, we considered the RIFLE criteria to be the most suitable for a review of renal function in experimental porcine sepsis. RIFLE is based on relative changes in serum creatinine or GFR, as well as body weight adjusted urine output. The modifications of the RIFLE criteria introduced to the later AKIN and KDIGO criteria do not appear to be immediately applicable to animal studies, in which baseline measurements are usually well defined. The introduction of an absolute change in serum creatinine as a diagnostic criterion was based on associations with mortality in human populations. The applicability of this threshold to other species, however, remains uncertain. Consensus derived relative changes in kidney function, as emphasized by the original RIFLE criteria – and still the backbone of the KDIGO criteria - are more likely appropriate. Furthermore, the omission of the GFR criteria in the AKIN and KDIGO criteria is unfortunate in experimental settings, as measurement of GFR is frequently performed in porcine models. The KDIGO group acknowledges GFR as the most useful overall index of kidney function (Kellum et al., 2012), and use of precise GFR markers may further enhance the relevance of GFR measurements in critical illness (Dixon et al., 2018). The urine output threshold used in the RIFLE criteria (<0.5 mL/kg/h) also appears biologically relevant in pigs, as reported normal urine output in healthy pigs is above this level (0.6-9.6 mL/kg/h) (Hannon et al., 1990). Taken together, although the RIFLE criteria have not been formally validated in pigs, they provide a pragmatic and transparent framework for standardized assessment of renal dysfunction across heterogenous porcine sepsis studies.

Although renal biopsies are considered a gold standard for diagnosis and characterization of kidney injury in many renal diseases, structural assessment does not reflect the degree of renal functional impairment in sepsis. In both animal models and human sepsis, severe reductions in glomerular filtration rate and overt AKI, when defined by functional criteria, often occur in the absence of widespread tubular cell death or overt structural damage on light microscopy. Biopsies in septic patients have shown that, despite a high prevalence of AKI defined by consensus criteria, the majority of renal tubular cells in most patients appear morphologically intact (Takasu et al., 2013). Sheep subjected to E. coli-induced septic shock developed oliguria, increased serum creatinine, and reduced creatinine clearance consistent with AKI, while renal biopsies revealed minimal structural alterations (Maiden et al., 2016). The only histological difference between septic and non-septic animals was mesangial expansion detectable on electron microscopy. In the context of porcine sepsis models, functional measurements therefore represent the most relevant and translationally meaningful standard for the assessment of AKI.

The aim of this review was to assess the renal function in untreated porcine models of sepsis using the RIFLE criteria at the group level. We therefore designed a systematic review with RIFLE-based classification of AKI as the primary outcome measure. Furthermore, we also aimed to report on which specific steps of the RIFLE criteria (Risk, Injury, Failure) that were fulfilled, and which parameters that were used (e.g., serum creatinine, urine output). We aimed to analyze whether the incidence of AKI varied depending on the method used to induce sepsis. A secondary aim was to examine whether precise exogenous markers of kidney function had been applied in porcine models of sepsis (e.g. inulin, iohexol, iothalamate, Cr-EDTA). We also aimed to report if AKI/ARF was reported by the authors, and if so whether the claim was supported by a defined criterion or classification system. Lastly, we wanted to document which definitions of AKI/ARF that had been reported.

## Methods

### Search protocol

This review was pre-registered in the Open Science Framework (Registration DOI: 10.17605/OSF.IO/FC29M). The Preferred Reporting Items for Systematic Reviews and Meta-Analyses (PRISMA 2020) checklist was used as a guide for reporting the review (Page et al., 2021).

### Search strategy

**Figure 1:**
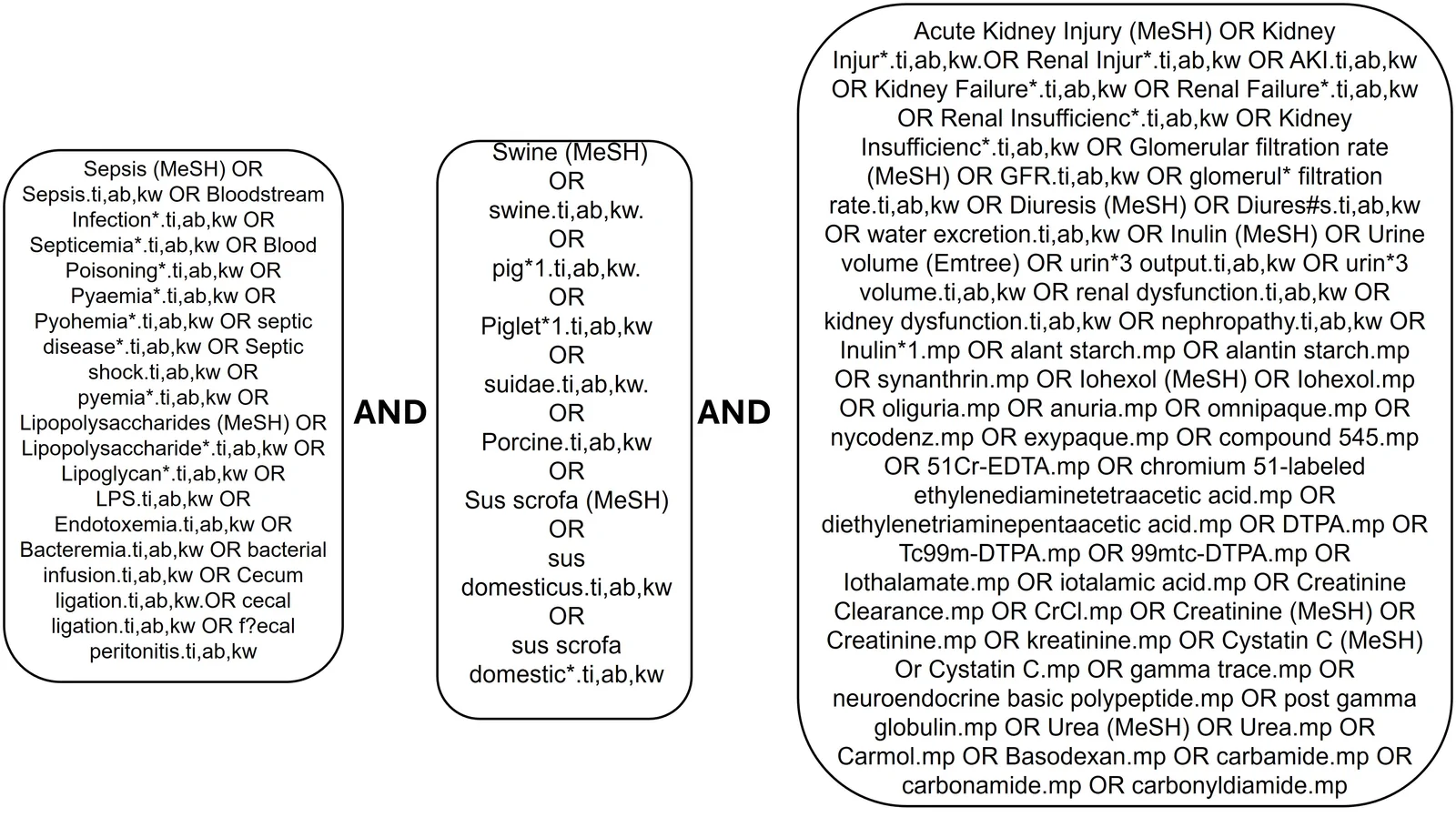
Literature search strategy. The literature search strategy used a building block strategy with Boolean operators. The above search strategy shows how the search was performed in Ovid Medline and Ovid Embase.

The search method used a building block strategy with Boolean operators. A comprehensive literature search strategy was developed with the participation of all authors, encompassing the period from 1946 to April 29^th^, 2025 (**Figure 1**). The following databases were searched: Ovid MEDLINE(R) and Epub Ahead of Print, In-Process, In-Data-Review & Other Non-Indexed Citations, Daily and Versions, Ovid Embase Classic + Embase, and Clarivate™ (Web of Science™) ^©^ Clarivate 2024™. No automatic filtering option was applied. Additionally, a manual examination of the reference lists of the retrieved eligible articles was conducted.

The search strategy was in accordance with PRISMA systematic review guidelines (Page et al., 2021). We used the controlled vocabulary of Medical Subject Headings (MeSH) from MEDLINE and the Emtree thesaurus from EMBASE, where applicable. Furthermore, free-text words were used to search Titles, Abstracts, and Authors keywords fields, in all three databases. For most of the pharmacological search words in Medline and EMBASE, we used the search field.mp (title, book title, abstract, original title, name of substance word, subject heading word, floating sub-heading word, keyword heading word, organism supplementary concept word, protocol supplementary concept word, rare disease supplementary concept word, unique identifier, synonyms, population supplementary concept word, anatomy supplementary concept word). The complete search strategy from Ovid MEDLINE is provided as **Figure 1**, and a screenshot of the Ovid MEDLINE search is provided as a **Supplementary Figure**.

### Screening, study selection and eligibility criteria

All retrieved references were deduplicated and screened in two stages (title/abstract and full text) by independent reviewers working in pairs, using predefined inclusion and exclusion criteria. Prior to formal screening, a calibration exercise was conducted to ensure consistency and reproducibility among screeners. Disagreement was resolved by consensus, with unresolved conflicts proceeding to full-text assessment. A detailed description of the screening process, study selection, and eligibility criteria is provided in the **Supplementary Methods**. Study selection criteria are outlined in **Table 2**.

**Table 2:** Study selection criteria.

Inclusion criteria
*In vivo*, porcine study
Terminal study design, maximum 24h duration
Aim of creating sepsis or sepsis like conditions
Report data on changes in glomerular filtration rate marker or urinary output from baseline in at least one group that did not receive antimicrobial treatment, anti-inflammatory treatment, immunomodulatory therapy, nephrotoxic doses of pharmacological agents, extracorporeal membrane oxygenation, renal replacement therapy (i.e. hemodialysis and/or hemofiltration) and/or plasmapheresis
Scandinavian or English language
Peer-review studies
Exclusion criteria
Pathology in the extrarenal urinary system
Surgery involving the renal parenchyma or renal vasculature (except for placement of microdialysis catheters and flow probes)
Sepsis induced by pathogens introduced directly into the urinary system

Inclusion and exclusion criteria applied for study selection in the review.

### Data extraction and synthesis

Data extraction was performed independently by two authors, with discrepancies resolved by consensus involving a third author. Importantly, the selected studies were classified according to whether RIFLE criteria were fulfilled or not. Studies which fulfilled the RIFLE criteria were subsequently classified according to the risk, injury and failure criteria. RIFLE staging was performed by applying either the mean or the median data from the group in question, depending on what was available. A detailed description of the data extraction process and outcome classification is provided in the **Supplementary Methods**.

### RIFLE interpretation and AKI

The term AKI was not yet been established when the RIFLE criteria were introduced. With the exception of the GFR component, the creatinine and urine output thresholds for RIFLE Risk, Injury and Failure largely corresponds with AKI stage 1, 2 and 3 of the KDIGO criteria (Kellum et al., 2012). Accordingly, for the purpose of this review, fulfilment of RIFLE Risk or greater was considered diagnostic of AKI.

### Statistics

Statistical analyses were conducted at the study (group) level using predefined binary RIFLE outcomes, with descriptive statistics summarizing study characteristics and RIFLE fulfilment. Associations between reported renal parameters, claimed renal dysfunction, and RIFLE fulfilment were examined using Fisher´s exact test and study-level logistic regression, with results expressed as odds ratio and results considered statistically significant at *p-*values <0.05. Full details of the statistical analyses are provided in the **Supplementary Methods**.

### Meta-analysis

A random-effects meta-analysis of study-level proportions was performed to estimate the pooled proportion of studies fulfilling at least RIFLE Risk, with proportions analysed on the logit scale (Barker et al., 2021). Subgroup analyses were conducted by sepsis induction method, and sensitivity analyses explored the impact of study size. A complete description of the meta-analytic methods and sensitivity analyses is available in the **Supplementary Methods**.

## Results

### Literature search results

**Figure 2:**
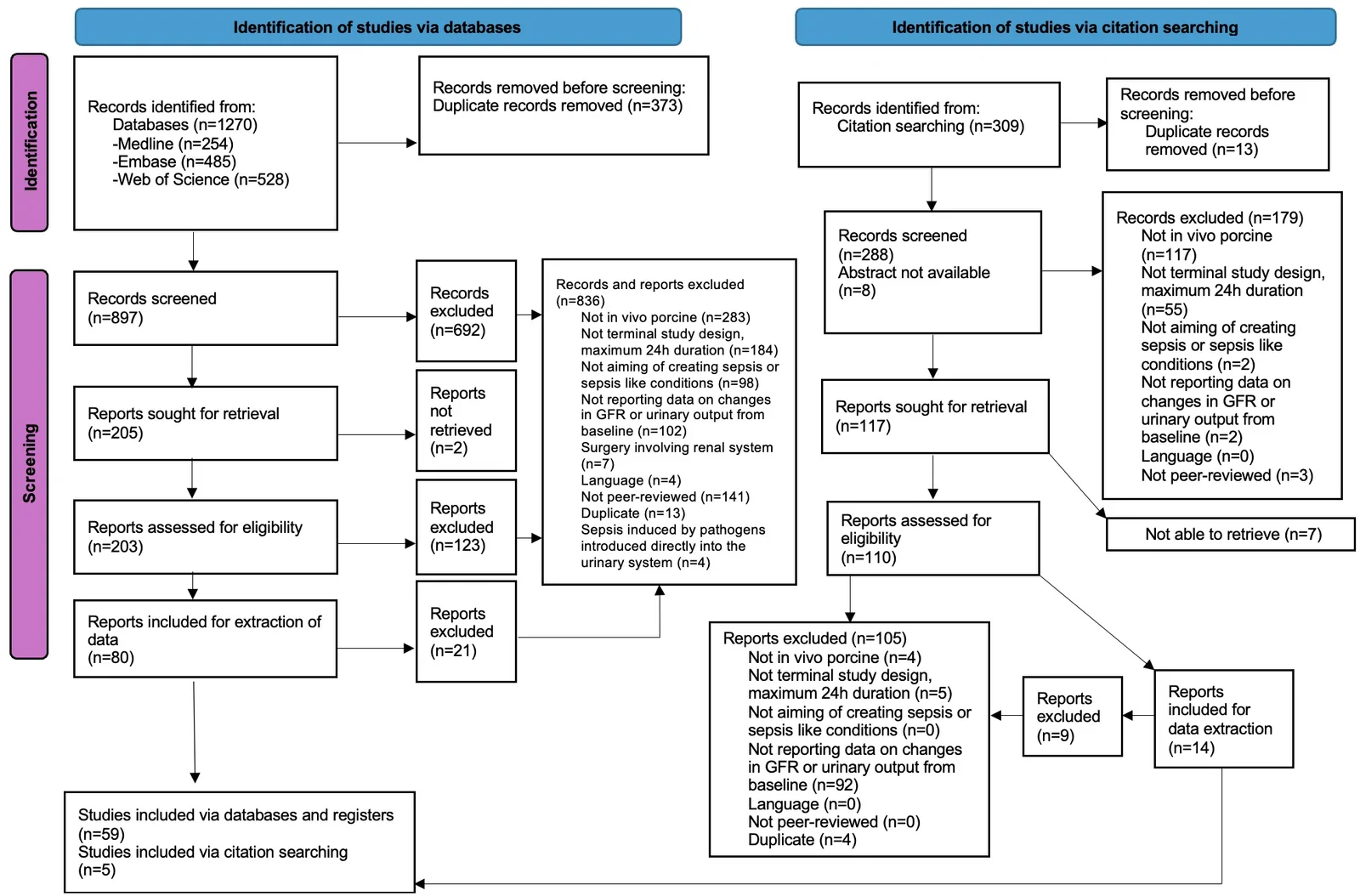
Prisma 2020 flow diagram. A flow diagram detailing the search, screening and data extraction process. This figure is based on the PRISMA 2020 flow diagram template, which is distributed in accordance with the terms of the Creative Commons Attribution license (Page et al., 2021).

The PRISMA 2020 flow diagram is shown in **Figure 2**. 1270 records were retrieved via databases and registers. 897 records were screened after removal of duplicates. After screening of titles, abstracts and full text articles, 59 studies were included for review. Additional 309 records were identified from citation searching. After screening of titles, abstracts and full text articles, 5 studies were included for review. The most common reasons for exclusion during the screening process were “not *in vivo* porcine studies”, “experimental duration >24h”, and “not peer reviewed studies”.

Accordingly, a total of 64 studies were included in the current systematic review.

### Study characteristics

All studies were categorized according to which RIFLE criteria they fulfilled at the group level. 30 of 64 (47%) studies fulfilled one of the RIFLE criteria, of which 16 fulfilled the Risk criteria, nine the Injury criteria, and five the Failure criteria. 34 (53%) studies did not fulfil any of the RIFLE criteria. 45 (70%) of the included studies reported change in creatinine from baseline, 33 (52%) reported urine output and 15 (23%) reported change in GFR from baseline.

All included studies were published between 1984 and 2025. Number of animals included in each study varied considerably. For RIFLE positive studies, five studies were performed in piglets weighing 1.9–4 kg, while the remaining studies utilized pigs weighing 18.3–74 kg. Of the 30 studies which fulfilled RIFLE, only one study reported all three RIFLE criteria parameters (creatinine, urine output, and GFR). Precise methods for GFR measurements were not applied in any of the studies which did not fulfil the RIFLE criteria. Only three RIFLE positive studies applied a precise exogenous marker for GFR monitoring. Chin et al. and Lievano et al. used continuous infusion of iothalamate in a study in which the Risk and Injury criteria were fulfilled, respectively (Lievano et al., 1998; Chin et al., 2002). Sølling et al. applied ^51^Cr-EDTA in a study where the RIFLE Injury criteria was fullfilled (Solling et al., 2011).

In total, nine studies applied intravenous (IV) Escherichia coli (E. coli), four applied IV Pseudomonas aeruginosa (P. aeruginosa), 33 studies applied IV lipopolysaccharide (LPS), 17 studies applied fecal peritonitis and one study applied Group A Streptococci. The most frequently used method for sepsis induction across all studies which met one of the RIFLE criteria was infusion of LPS. This method was applied in 19 out of the 30 studies. IV infusion with live P. aeruginosa bacteria was used in three studies and IV infusion of live E. coli bacteria in another three studies. Fecal peritonitis was applied to induced sepsis in five of the RIFLE positive studies. Studies in which sepsis was induced by fecal peritonitis lasted typically 12–24 h, while intravenous induced sepsis was terminated typically after 2–12 h. Summarized key characteristics of porcine sepsis models in which the RIFLE Risk, Injury and Failure criteria are fulfilled are provided in **Tables 3**–**5**, respectively. Summarized key characteristics of porcine sepsis models in which the RIFLE criteria were not fulfilled are provided in **Tables 6**–**8**. Further descriptive details of selected studies which fulfilled the RIFLE criteria are included in the Supplemental Results.

**Table 3:** Characteristics of experimental porcine sepsis models which fulfill the RIFLE risk criteria.

Reference	Year	Animal strain	Number of animals included	Weight (kg)	Method for sepsis induction			Length of study	Creatinine reported	Urine output reported	GFR reported
					Live bacteria infusion	LPS infusion	Fecal peritonitis				
Rokke et al. (1989)	1989	NA	7	23-33	Escherichia coli			8 h	No	Yes	No
Eriksson et al. (1998)	1998	NA	9	23-30		Escherichia coli		6 h	Yes	No	No
Chin et al. (2002)	2002	Domestic piglets	5	2.5-3.0		Escherichia coli		3 h	No	Yes	Iothalamate
Wagner et al. (2002)	2002	Yorkshire pigs	8	31 ± 4			Cecal content + Escherichia coli	15 h	Yes	Yes	No
Matejovic et al. (2005)	2005	Domestic pigs	8	20-25	Pseudomonas aeruginosa			24 h	Yes	No	No
Mazzola et al. (2005)	2005	White pigs	4	22.0 ± 2.7		Escherichia coli		4 h	Yes	No	No
Barth et al. (2008)	2008	Domestic pigs	20	46–59			Autologous feces	24 h	Yes^*^	No	Creatinine clearance
Furtado et al. (2008)	2008	Neonatal farm pigs	7	2.2-4.0		Escherichia coli		3h	No	Yes	GFR mL/min/g
John et al. (2008)	2008	Neonatal farm pigs	5	2.3-2.7		Escherichia coli		3h	No	No	GFR mL/min
Duburcq et al. (2014)	2014	White pigs	5	NA		Escherichia coli		5 h	Yes	Yes	No
Laroye et al. (2018)	2018	Domestic pigs	6	40-60			Autologous feces	24 h	Yes	No	No
Lima et al. (2018)	2018	Crossbred Landrace × Yorkshire	9	28.2 ± 1.9		Escherichia coli		NA	Yes	Yes	No
Izquierdo-Garcia et al. (2019)	2019	NA	6	27± 4	Escherichia coli			300 min	Yes	Yes	No
Sallustio et al. (2019)	2019	Domestic pigs	NA	45–55		Escherichia coli		24 h	Yes	No	No
Fanous et al. (2022)	2022	Domestic piglets	7	2.5-3.5			Cecal puncture	12 h	Yes	No	No
Kurita et al. (2024)	2024	Domestic pigs	13	22-29.5		Escherichia coli		4 h	Yes	No	No

*: Creatinine concentrations normalized per gram of plasma protein. RIFLE, Risk, Injury, Failure, Loss, End-stage kidney disease; NA, No information available; LPS, Lipopolysaccharide; GFR, Glomerular filtration rate.

**Table 4:** Characteristics of experimental porcine sepsis models which fulfill the RIFLE injury criteria.

Reference	Year of publication	Animal strain	Number of animals included	Weight (kg)	Method for sepsis induction			Length of study	Creatinine reported	Urine output reported	GFR reported
					Live bacteria infusion	LPS infusion	Fecal peritonitis				
Lievano et al. (1998)	1998	Domestic pigs	15	1.9-2.5		Escherichia coli		3 h	No	No	Iothalamate
Martinez et al. (2001)	2001	Yorkshire pigs	5	31 ± 4			Cecal content + Escherichia coli	8 h	Yes	Yes	No
Lipcsey et al. (2005)	2005	Domestic pigs	6	26.4-29.2	Escherichia coli			6 h	No	No	Creatinine clearance
Fenhammar et al. (2011)	2011	Landrace/ Yorkshire/Hampshire	8	33.3 ± 0.7		Escherichia coli		5 h	No	No	Creatinine clearance
Solling et al. (2011)	2011	Landrace/ Yorkshire/ Duroc pigs	10	31-35		Escherichia coli		10 h	Yes	No	^51^Cr-EDTA
Granfeldt et al. (2014)	2014	Landrace/ Yorkshire/ Duroc pigs	8	37-42		Escherichia coli		5 h	Yes	Yes	Creatinine clearance
Stasi et al. (2023)	2023	Domestic pigs	6	NA		Escherichia coli		24 h	Yes	Yes	No
Olney et al. (2024)	2024	Outbred Yorkshire	4	70-74		Escherichia coli		350 min	Yes	Yes	No
Sugita et al. (2024)	2024	Landrace × Large White × Duroc	5	29-35.3		Escherichia coli		4 h	Yes	No	No

RIFLE, Risk, Injury, Failure, Loss, End-stage kidney disease; NA, No information available; LPS, Lipopolysaccharide; GFR, Glomerular filtration rate.

**Table 5:** Characteristics of experimental porcine sepsis models which fulfill the RIFLE failure criteria.

Reference	Year of publication	Animal strain	Number of animals included	Weight (kg)	Method for sepsis induction			Length of study	Creatinine reported	Urine output reported	GFR reported
					Live bacteria infusion	LPS infusion	Fecal peritonitis				
Wang et al. (2013)	2013	Minipigs	6	18.7± 1.2		Escherichia coli		24 h	Yes	Yes	No
Vassal et al. (2015)	2015	Landrace pigs	6	20-25	Pseudomonas aeruginosa			160 min	No	Yes	Creatinine clearance
von Seth et al. (2015)	2015	Domestic pigs	6	22.3-29.3		Escherichia coli		6 h	No	No	Creatinine clearance
Duburcq et al. (2018)	2018	White pigs	5	18.3-24.0		Escherichia coli		5 h	No	Yes	Creatinine clearance
Hong et al. (2025)	2025	Yuna breed	12	55-60	Pseudomonas aeruginosa			24 h	Yes	Yes	No

RIFLE, Risk, Injury, Failure, Loss, End-stage kidney disease; LPS, Lipopolysaccharide; GFR, Glomerular filtration rate.

**Table 6:** Characteristics of bacteria induced porcine sepsis models in which RIFLE criteria were not fulfilled.

Reference	Year of publication	Animal strain	Bacteria	Number of animals included	Weight (kg)	Length of study	Creatinine reported	Urine output reported	GFR reported	Comments
Hardaway et al. (1996)	1996	NA	Escherichia coli	8	27-32	12h	No	Yes	No	Urine output >0.5 mL/kg/h
Saetre et al. (2000)	2000	NA	Group A Streptococci	6	21-32	5h	Yes	Yes	No	<6 h experiment Creatinine increased <1.5x baseline
García-Septien et al. (2010)	2010	NA	Escherichia coli	7	25-35	6h	Yes	Yes	No	Urine data for only 5 h Creatinine<1.5 x baseline
Otero et al. (2014)	2014	Swedish landrace	Escherichia coli	8	27 ± 4	6h	No	Yes	No	Urine output >0.5 mL/kg/h
Matejovic et al. (2017)	2017	NA	Pseudomonas aeruginosa	2	NA	22h	Yes	No	No	Creatinine<1.5 x baseline
Toth et al. (2017)	2017	Hungahib pigs	Escherichia coli	10	19.5 ± 1.6	4h	Yes	No	No	Creatinine<1.5 x baseline
Von Seth et al. (2017)	2017	Domestic pigs	Escherichia coli	32	24 ± 2	6h	No	Yes	No	Urine output >0.5 mL/kg/h
Skorup et al. (2020)	2020	Landrace-breed	Escherichia coli	4	24.7 ± 1.4.	6h	Yes	Yes	No	Creatinine<1.5 x baseline Urine output >0.5 mL/kg/h

RIFLE, Risk, Injury, Failure, Loss, End-stage kidney disease; NA, No information available; GFR, Glomerular filtration rate.

**Table 7:** Characteristics of LPS induced porcine sepsis models in which RIFLE criteria were not fulfilled.

Reference	Year of publication	Animal strain	Number of animals included	Weight (kg)	Length of study	Creatinine reported	Urine output reported	GFR reported	Comments
Andersen et al. (1984)	1984	White landrace pigs	7	25-40	6h	No	Yes	No	Urine output >0.5 mL/kg/h
Lindsetmo et al. (1994)	1993	NA	7	18-33	8h	No	Yes	No	Urine output >0.5 mL/kg/h
Siebeck et al. (1994)	1994	Miniature pigs	10	18-33	8h	Yes	No	No	Creatinine<1.5 x baseline
Hauser et al. (2005)	2005	German landrace	8	24 (mean)	24h	Yes*	Yes	No	Urine output >0.5 mL/kg/h Creatinine normalized
Lipcsey et al. (2008)	2007	Triple breed pigs	18	27 ± 0.6	6h	No	Yes	No	Urine output >0.5 mL/kg/h
Fenhammar et al. (2008)	2008	Landrace/ Yorkshire/Hampshire	8	30-36	5h	Yes	Yes	No	Creatinine<1.5 x baseline Urine output >0.5 mL/kg/h
Carlsson et al. (2009)	2009	Healthy piglets	24	26.9± 2.4	6h	Yes	No	No	Creatinine<1.5 x baseline
Lipcsey et al. (2010)	2010	Domestic breed pigs	19	23-31	6h	No	Yes	No	Urine output >0.5 mL/kg/h
Chew et al. (2011)	2011	Landrace pigs	8	22-35	6h	Yes	No	No	Creatinine<1.5 x baseline
Kristensen et al. (2011)	2011	Landrace pigs	10	20-25	3h	No	Yes	Yes	GFR<25% decreased
Söderberg et al. (2012)	2012	Domestic breed pigs	4	25.5-28.9	6h	Yes	No	Yes	GFR<25% decreased
Sperber et al. (2013)	2013	Healthy pigs	10	25.8 ± 1.6	7h	Yes	No	No	Creatinine<1.5 x baseline
Strandberg et al. (2014)	2014	Domestic pigs	8	21.2-29.1	6h	Yes	No	No	Creatinine<1.5 x baseline
Gomez et al. (2021)	2021	Yorkshire-duroc	14	27.2-36.4	5h	Yes	No	No	Creatinine<1.5 x baseline

*Creatinine concentrations normalized per gram of plasma protein. RIFLE, Risk, Injury, Failure, Loss, End-stage kidney disease; NA, No information available; LPS, Lipopolysaccharide; GFR, Glomerular filtration rate.

**Table 8:** Characteristics of fecal peritonitis induced porcine sepsis models where the RIFLE criteria were not fulfilled.

Reference	Year of publication	Animal strain	Number of animals included	Weight	Length of study	Creatinine reported	Urine output reported	GFR reported	Comments
Strand et al. (1998)	1998	Domestic pigs	9	21–33 kg	9 h	No	Yes	No	Urine output >0.5 mL/kg/h
de Azevedo et al. (2007)	2007	White large pigs	2	40–45 kg	24 h	Yes	Yes	No	Urine output >0.5 mL/kg/h Creatinine<1.5 x baseline
Chvojka et al. (2008)	2008	Domestic pigs	6	27–35 kg	22 h	Yes	Yes	No	Urine output >0.5 mL/kg/h. Creatinine<1.5 x baseline
Simon et al. (2009)	2009	Pigs	12	38–61 kg	24 h	No	Yes	Yes	<25% decrease in GFR Urine output >0.5 mL/kg/h
Sykora et al. (2009a)	2009	Domestic pigs	7	34–42 kg	22 h	Yes	No	No	Creatinine<1.5 x baseline
Sykora et al. (2009b)	2009	Domestic pigs	8	32–38 kg	22 h	Yes	No	No	Creatinine<1.5 x baseline
Kozlov et al. (2010)	2010	Domestic pigs	7	25–30 kg	12 h	Yes	No	No	Creatinine<1.5 x baseline
Goto et al. (2012)	2012	Mixed-strain piglets	6	1646 ± 88 gram	6 h	Yes	No	No	Creatinine<1.5 x baseline
Soni and Adebiyi (2017)	2017	Neonatal piglets	7	*3–5 days old*	6 h	Yes	No	No	Creatinine<1.5 x baseline
Park et al. (2019)	2019	Yorkshire-X domestic pigs	11	49 ± 8 kg	12 h	Yes	Yes	No	Urine output >0.5 mL/kg/h. Creatinine<1.5 x baseline
Ferrario et al. (2019)	2019	Domestic pigs	6	42,3 ± 3.9 kg	NA	Yes	No	No	Creatinine<1.5 x baseline
Pinto et al. (2022)	2022	Domestic pigs	6	42.3 ± 3.9 kg	6 h	Yes	No	No	Creatinine<1.5 x baseline

RIFLE, Risk, Injury, Failure, Loss, End-stage kidney disease; GFR, Glomerular filtration rate.

### Statistics

28 out of 64 (44%) studies claimed renal dysfunction. Only two of these applied consensus-based criteria (AKIN & KDIGO) (Matejovic et al., 2017; Gomez et al., 2021), and none applied porcine specific AKI criteria. In univariable logistic regression, claimed renal dysfunction was associated with higher odds of RIFLE-defined AKI; however, this association did not reach statistical significance (OR 2.1, *p* = 0.15).

We evaluated whether the renal function parameter used (creatinine, urine output, GFR) was associated with fulfilment of at least RIFLE Risk (**Table 9**). Among studies reporting creatinine, 44% fulfilled at least RIFLE Risk (20 of 45), compared with 10 of 19 (53%) amongst studies not reporting creatinine (*p* = 0.59). Similarly, reporting of urine output was not associated with RIFLE fulfilment. Among studies reporting urine output, 15 of 33 (46%) fulfilled at least RIFLE Risk, compared with 15 of 31 (48%) among studies not reporting urine output (*p* = 1.00). In contrast, reporting of GFR was strongly associated with fulfilment of RIFLE criteria. Among studies reporting GFR, 12 of 15 (80%) fulfilled at least RIFLE Risk, compared with 18 of 49 (37%) among studies not reporting GFR (*p* = 0.007). Applying logistic regression, reporting of GFR was associated with significantly higher odds of RIFLE fulfilment (OR 6.9, 95% CI: 1.7-27.7; *p* = 0.007). There was no significant association between reporting multiple renal parameters and fulfilling at least the RIFLE Risk stage (*p* = 0.13).

**Table 9:** Association between reported renal parameters and RIFLE classification assessed by study-level logistic regression.

Parameter reported	RIFLE ≥risk (%)	Odds ratio	95% CI	*P*-value
Creatinine	44%	0.72	0.25-2.11	0.55
Urine output	46%	0.89	0.33-2.38	0.81
GFR	80%	6.9	1.71-27.72	0.007
≥2 renal parameters	59%	2.0	0.81-5.04	0.13

Renal parameters were defined as the number of reported kidney-related measures (serum creatinine, urine output, and GFR) per study.

RIFLE, Risk, Injury, Failure, Loss, End-stage kidney disease; GFR, Glomerular filtration rate.

### Meta-analysis

**Figure 3:**
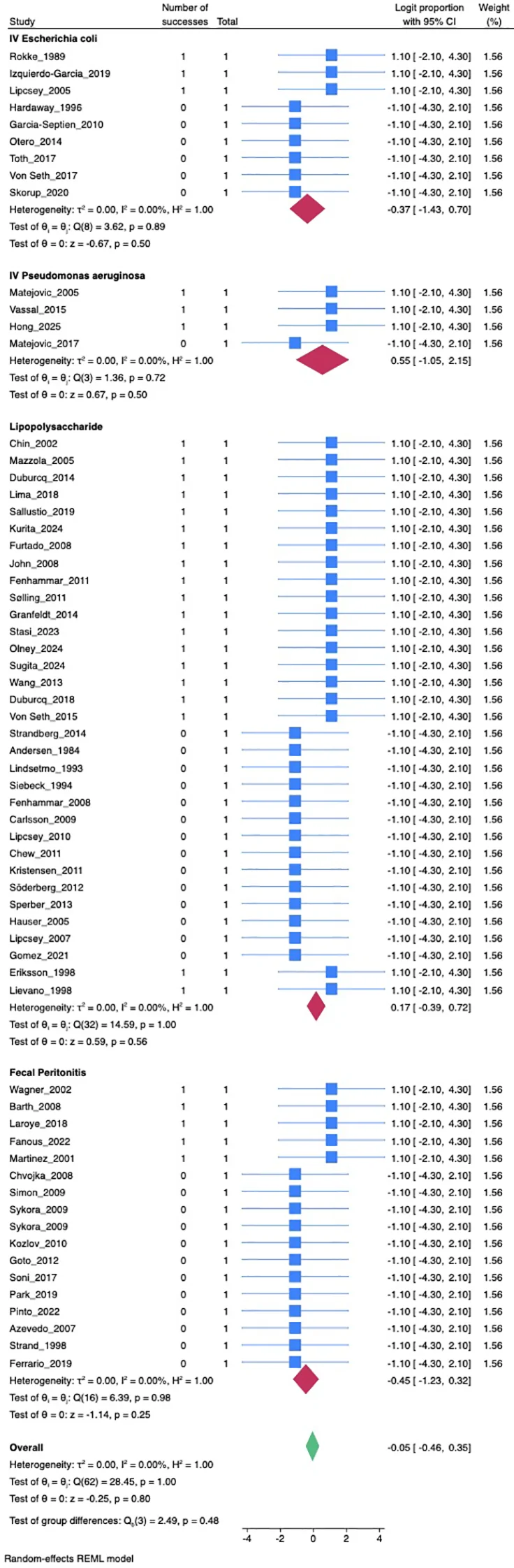
Forest plot. Forest plot of study-level proportions of experimental porcine sepsis studies meeting versus not meeting at least the RIFLE Risk criteria, stratified by sepsis induction method.

Data from the meta-analyses are presented in **Table 10**. In the random-effects meta-analysis of proportions, the pooled estimate indicated that 48% of studies fulfilled at least one RIFLE criterion (95% CI 39–58%). Subgroup analyses stratified by sepsis induction method showed similar proportions of RIFLE fulfilment across models, with wide confidence intervals (**Figure 3**). The pooled proportion of RIFLE-positive studies was 41% (95% CI 19–67%) for live E. coli, 63% (95% CI 26–90%) for live P. aeruginosa, 54% (95% CI 40–67%) for LPS and 39% (95% CI 23–58%) for fecal peritonitis. No statistically significant differences between sepsis induction methods were found (Q_between = 2.49, *p* = 0.48).

**Table 10:** Meta-regression and heterogeneity diagnostics.

Model/Moderator	k (studies)	β	SE	*p*-value	95% CI
Overall meta-analysis (no covariates)	64	0.48*	–	0.74	0.39* to 0.58*
Meta-regression: number of animals	63	-0.038	0.041	0.35	-0.12 to 0.04
Meta-regression: √(number of animals)	63	-0.240	0.278	0.39	-0.79 to 0.31
Meta-regression: sepsis induction method + number of animals	62	–	–	0.51**	–
Meta-regression: sepsis induction method + √(number of animals)	62	–	–	0.53**	–

Random-effects meta-regression analyses were conducted using restricted maximum likelihood estimation. Effect sizes were estimated on the logit scale. When relevant, these are presented as back-transformed proportions with corresponding 95% confidence intervals; back-transformed proportions are marked with *.β, meta-regression coefficient; SE, standard error; **Global Wald test for categorical moderator (sepsis induction method).

In sensitivity analyses using random-effects meta-regression, study size was not associated with fulfilment of RIFLE criteria. Neither the number of animals (regression coefficient (β) = −0.038, 95% CI −0.12 to 0.04; *p* = 0.35) nor the square root–transformed number of animals (β = −0.24, 95% CI −0.79 to 0.31; *p* = 0.39) showed a significant association with RIFLE classification. Furthermore, adjustment for number of animals did not materially alter the association between sepsis induction method and RIFLE fulfilment. Compared with live E. coli as the reference method, none of the alternative sepsis induction methods were associated with a significantly different effect estimate (P. aeruginosa: β = 0.80, *p* = 0.42; LPS: β = 0.47, *p =* 0.45; fecal peritonitis: β = -0.17, *p* = 0.81). Between-study heterogeneity as quantified by I² was negligible throughout all analyses (I^2^ = 0.00); however, conventional heterogeneity metrics are of no value given the binary study-level classification and the use of logit-transformed proportions.

## Discussion

Sepsis is the most common cause of AKI, and SA-AKI is associated with a poor prognosis, with a substantial proportion of patients progressing to stage 3 AKI (Ostermann et al., 2025). Despite significant discoveries the pathogenesis of SA-AKI remains elusive. Porcine models have been increasingly utilized to investigate pathophysiological mechanisms (Huang et al., 2021). To our knowledge, no review existed on renal function in porcine models of sepsis. Hence, we aimed to interrogate and close this knowledge gap by performing a systematic review and meta-analysis. We found that almost half of the included studies fulfilled at least RIFLE Risk at the group level. Reporting of GFR, but not serum creatinine or urine output, was consistently associated with a higher likelihood of fulfilling at least RIFLE Risk at the group level. Furthermore, the meta-analysis did not identify a statistically robust association between different sepsis induction methods and RIFLE fulfilment. However, this finding should be interpreted with considerable caution, as heterogeneous study designs, large variations in experimental methodology, and inconsistent reporting limited both the comparability of studies and the interpretability of the pooled analyses.

Renal impairment was claimed in 44% of the included studies, but only two studies based this claim upon a defined criteria for AKI (Matejovic et al., 2017; Gomez et al., 2021). This is not entirely unexpected, because the primary aim in most studies was not to study SA-AKI pathophysiology, and many studies have reported statistically significant differences in a renal function parameter either from baseline or compared to control animals. Nevertheless, the lack of broadly applied porcine specific AKI criteria is unfortunate as interpretation of translation research depends very much on a common vocabulary and same set of definitions. The presence of author-reported renal dysfunction was ultimately not significantly associated with fulfilment of RIFLE criteria for AKI when assessed at a group level, supporting the need for defined criterion for AKI diagnosis in porcine models.

The findings from the subgroup meta-analyses suggests that the likelihood of fulfilling RIFLE criteria at the group level is not primarily determined by the sepsis induction method itself. This may indicate that none of these four primary methods (E. coli infusion, P. Aeruginosa infusion, LPS infusion and fecal peritonitis) are inherently superior if the sole aim is to induce porcine SA-AKI within a 24-hour timeframe. However, given the limited number of included studies in each group, substantial methodological heterogeneity, and the assessment of RIFLE fulfilment based on aggregated data, these findings should be interpreted with considerable caution. Moreover, the extent of AKI induction alone does not necessarily determine the suitability of a model for studying AKI pathophysiology or treatment, as mechanisms of renal injury may vary across experimental models, among patients with SA-AKI, and between experimental models and clinically occurring human SA-AKI.

Instead, the present data suggest that the diagnosis of AKI according to RIFLE criteria in experimental sepsis studies is influenced by the method used to assess renal function. This may reflect the sensitivity of the individual components of the RIFLE criteria themselves, as well as limitations of specific renal function parameters. Urine output criteria require sustained oliguria and is therefore problematic in studies with short observation periods. Consequently, when urine output is the sole marker of renal function, RIFLE Injury and Failure are difficult to meet in short-duration experiments, in contrast to creatinine- and GFR-based criteria that rely on relative changes. Regarding creatinine, the increase in serum concentration that is expected in AKI has been demonstrated to be blunted due to decreased production in sepsis (Doi et al., 2009). These methodological considerations, together with this review’s finding that reporting of GFR was associated with a higher likelihood of fulfilling RIFLE criteria, have implications for future studies of SA-AKI. Direct measurement of GFR should be considered mandatory. Precise markers of GFR have been demonstrated to be more sensitive for alterations in renal function than renal parameters such as creatinine in dynamic physiological states in humans (Dixon et al., 2018; Jakobsen et al., 2022). However, their use remains limited not only in experimental studies, but also in human research within this context. The near absence of precise markers in the included studies, together with the finding that RIFLE fulfillment is associated with GFR being reported, further strengthens the rationale for their broader implementation in future studies. Stress and damage biomarkers such as L-FABP and TIMP2 may also provide important complementary information (Ostermann et al., 2024). While alternative markers have gained increased attention in recent years, their use remain inconsistent, and standardized thresholds for staging AKI are often based on absolute values and have not been validated across species (Ostermann et al., 2024). Thus, we chose not to include biomarker-based criteria in the present review. Biological sex may influence renal physiology and susceptibility to SA-AKI (Curtis, 2024). The frequent use of animals of only one sex, or failure to report sex, represents a broader weakness in experimental sepsis research and should receive greater attention in future studies. Furthermore, current functional criteria such as RIFLE, AKIN and KDIGO does not account for sex differences. Whether such thresholds would improve AKI classification represents an area of future investigation.

We anticipated that most articles would report creatinine, GFR or urinary output at a group level, and not at the individual level. This was confirmed in the review process. Two included studies explicitly reported that their animals fulfilled AKIN (Matejovic et al., 2017) or KDIGO criteria (Gomez et al., 2021) for acute kidney injury, yet did not fulfill RIFLE Risk when assessed in the current analysis. This discrepancy may reflect fundamental methodological differences. Firstly, differences between KDIGO, AKIN and RIFLE criteria may contribute to the differences seen. Furthermore, in those studies, AKI classification was based on individual animal data, such that urine output criteria could be met by one animal and serum creatinine criteria by another. The present analysis applied RIFLE criteria to aggregated values at the group level rather than individual animal data. As a result, within-study variability and individual-level extremes may have been attenuated when using mean/median values. This is an important limitation to the methodology of the current systematic review. However, this limitation was unavoidable, as individual animal data were rarely reported in the included studies, and retrieval of raw data from authors was considered unlikely to be feasible for many studies, particularly older publications. Applying RIFLE criteria at the group level is likely to reduce sensitivity for detecting AKI, as substantial renal dysfunction in individual animals may be masked by less affected animals within the same experimental group. Conversely, study groups that fulfilled RIFLE criteria necessarily demonstrated marked changes in mean or median renal function, suggesting that the specificity of this approach is relatively high. Accordingly, while this method cannot be used to determine the exact incidence of AKI within each study, it is likely to reflect the overall severity of renal dysfunction associated with each experimental model. Taken together, these considerations should be borne in mind when interpreting the results of this review. Nevertheless, the group-level application of a standardized AKI definition provided a pragmatic framework for comparing the renal impact of heterogeneous porcine sepsis models.

Inclusion criteria were developed iteratively to reduce heterogeneity in sepsis pathophysiology and enhance comparability between included studies. Only studies terminated within 24 hours were included, as this timeframe balances the slower development of AKI in fecal peritonitis models against the risk of bias induced by prolonged anesthesia and ventilation. Furthermore, limiting inclusion to studies with a similar study and sepsis duration improves the comparability between the included models. Included experiments were required to primarily induce sepsis or sepsis-like conditions without active treatment, excluding studies of sole localized infection, and to report changes in renal function from baseline in at least one untreated septic group (**Table 2**). As a result, several included groups originated from studies in which renal dysfunction was not the primary outcome. Sepsis induced renal effects may thus been underreported.

The conclusions of this review are supported by a rigorous methodology. We pre-registered and published a detailed protocol prior to the literature search, which was conducted across the major relevant databases. Screening and collection of data were performed by independent authors working pairwise, and a series of well-defined inclusion and exclusion criteria were applied. The systematic application of a standardized AKI definition across heterogenous experimental sepsis models represents another key strength to this review. The use of a meta-analytic framework provided a structured approach to summarize the available data and to explore potential sources of variability between studies. However, the binary nature of the data limited our ability to quantify statistical heterogeneity and reduced the power of subgroup and meta-regression analyses. Accordingly, these analyses should be considered exploratory and hypothesis generating. Nevertheless, in combination with the descriptive analyses, they provided additional insight into methodological factors that may influence fulfilment of AKI criteria.

Interpretation was complicated by the inconsistent reporting of renal function data across the included studies. Several screened reports did not have renal function as a primary outcome and failed to present renal data in a format that allowed assessment in the context of RIFLE. For example, several of the included articles reported data exclusively in graphical form (e.g. boxplots). Such studies were included only when the graphical data were sufficiently decisive to allow determination of RIFLE fulfilment at the group level. This inclusive approach enabled incorporation of a broader proportion of the existing literature than would have been possible had inclusion been restricted to studies reporting numerical renal function data. Despite these efforts, many studies were excluded due to insufficient reporting of renal function, which prevented assessment according to the RIFLE criteria. Studies published before the introduction of the RIFLE criteria in 2004 may have been particularly susceptible to exclusion, as reporting practices may have been less standardized. However, the core variables required for RIFLE classification - serum creatinine, GFR and urine output - have long been among the most commonly reported indicators of renal function in porcine models. We chose to also include studies published prior to the publication of the RIFLE criteria because they represent a substantial body of foundational work in porcine sepsis models. Excluding them would have omitted a large and relevant part of the available literature. Furthermore, these limitations would also apply to the AKIN and KDIGO classifications, and to an even greater extent, given their omission of the GFR-based criteria. Finally, although the RIFLE criteria have not been formally validated for use in porcine models, this limitation similarly applies to the other consensus-based AKI classifications. In the present review, use of an established human AKI definition was necessary to enable systematic comparison across heterogenous experimental sepsis models. However, this should not be interpreted as an endorsement of RIFLE as a validated tool for use in porcine research. Rather, the findings of this review highlight methodological factors that influence the detection of AKI across models and are intended to inform interpretation of existing studies. Ultimately, development of porcine-specific AKI classification systems would be expected to improve both internal validity and translational relevance of future studies on SA-AKI, and this represents an important area for further research.

## Conclusions

RIFLE-defined AKI at the group level was identified in 47% of untreated porcine sepsis studies. Exploratory meta-analysis did not identify statistically robust differences in RIFLE fulfilment between sepsis induction methods, but these findings were limited by small subgroup sizes, substantial methodological heterogeneity, and the use of aggregated study-level data. Reporting of GFR, but not serum creatinine or urine output, was associated with a higher likelihood of fulfilling at least RIFLE Risk at the group level, while precise markers remained infrequently used. Therefore, measurement of GFR should be incorporated into models of porcine sepsis. Furthermore, the development of porcine-specific AKI classification systems represents an important area for further research. The conclusions of this review provides novel insights that inform refinement of experimental designs and improve the translational value of future studies on SA-AKI.

## Data Availability

The raw data supporting the conclusions of this article will be made available by the authors, without undue reservation.
