# Supplementary material for: Does kidney function in experimental porcine sepsis fulfill the RIFLE criteria for acute kidney injury? A systematic review
Source: Front Physiol. 2026 Aug 19;17:1810924. doi: 10.3389/fphys.2026.1810924 (PMC13533698; doi:10.3389/fphys.2026.1810924)
Supplement: Supplementary file 1 [file DataSheet1.pdf]

Supplemental Figure. Screenshot of literature search performed in Ovid MEDLINE

#	Query	Results from 29 Apr 2025
1	exp Sepsis/	151,066
2	sepsis.ti,ab,kw.	135,908
3	bloodstream infection.ti,ab,kw.	8,599
4	septicemia*.ti,ab,kw.	16,493
5	blood poisoning.ti,ab,kw.	48
6	Pyaemia*.ti,ab,kw.	144
7	Pyohemia*.ti,ab,kw.	12
8	septic disease*.ti,ab,kw.	354
9	Septic shock.ti,ab,kw.	30,741
10	pyemia*.ti,ab,kw.	85
11	exp Lipopolysaccharides/	102,722
12	Lipopolysaccharide*.ti,ab,kw.	116,808
13	Lipoglycan*.ti,ab,kw.	253
14	LPS.ti,ab,kw.	116,805
15	Endotoxemia.ti,ab,kw.	8,929
16	Bacteremia.ti,ab,kw.	30,576
17	bacterial infusion.ti,ab,kw.	95
18	Cecum ligation.ti,ab,kw.	211
19	cecal ligation.ti,ab,kw.	5,971
20	f?ecal peritonitis.ti,ab,kw.	601
21	or/1-20	419,097
22	exp Swine/	254,612
23	swine.ti,ab,kw.	55,888
24	pig*1.ti,ab,kw.	241,199
25	piglet*1.ti,ab,kw.	24,614
26	suidae.ti,ab,kw.	262
27	porcine.ti,ab,kw.	102,876
28	exp Sus scrofa/	24,319
29	sus domesticus.ti,ab,kw.	82
30	sus scrofa domestic*.ti,ab,kw.	687
31	or/22-30	414,619
32	exp Acute Kidney Injury/	61,054
33	Kidney Injur*.ti,ab,kw.	54,372
34	Renal Injur*.ti,ab,kw.	16,016
35	AKI.ti,ab,kw.	26,607
36	Kidney Failure*.ti,ab,kw.	14,187
37	Renal Failure*.ti,ab,kw.	98,508
38	Renal Insufficienc*.ti,ab,kw.	25,953
39	Kidney Insufficienc*.ti,ab,kw.	803
40	exp Glomerular Filtration Rate/	53,333
41	GFR.ti,ab,kw.	22,967
42	glomerul* filtration rate.ti,ab,kw.	60,625
43	exp Diuresis/	21,354
44	Diures#s.ti,ab,kw.	14,203
45	water excretion.ti,ab,kw.	1,763
46	exp Inulin/	7,755
47	urin*3 output.ti,ab,kw.	9,640
48	urin*3 volume.ti,ab,kw.	9,214
49	renal dysfunction.ti,ab,kw.	22,773
50	kidney dysfunction.ti,ab,kw.	4,156
51	nephropathy.ti,ab,kw.	68,444
52	Inulin*1.mp.	13,439
53	alant starch.mp.	0
54	alantin starch.mp.	0
55	synanthrin.mp.	6
56	exp Iohexol/	5,326
57	Iohexol.mp.	6,409
58	oliguria.mp.	4,337
59	anuria.mp.	5,998
60	omnipaque.mp.	423
61	nycodenz.mp.	391
62	exypaque.mp.	2
63	compound 545.mp.	1
64	51Cr-EDTA.mp.	1,255
65	chromium 51-labeled ethylenediaminetetraacetic acid.mp.	12
66	diethylenetriaminepentaacetic acid.mp.	2,403
67	DTPA.mp.	23,778
68	Tc99m-DTPA.mp.	37
69	99mtc-DTPA.mp.	1,554
70	Iothalamate.mp.	1,685
71	iotalamic acid.mp.	3
72	Creatinine Clearance.mp.	20,349
73	CrCl.mp.	1,879
74	exp Creatinine/	64,430
75	Creatinine.mp.	168,187
76	kreatinine.mp.	7
77	exp Cystatin C/	5,241
78	Cystatin C.mp.	9,481
79	gamma trace.mp.	60
80	neuroendocrine basic polypeptide.mp.	1
81	post gamma globulin.mp.	12
82	exp Urea/	129,564
83	Urea.mp.	132,514
84	Carmol.mp.	1
85	Basodexan.mp.	1
86	carbamide.mp.	1,943
87	carbonamide.mp.	11
88	carbonyldiamide.mp.	4
89	or/32-88	696,517
90	21 and 31 and 89	255
