# Supplementary material for: Does kidney function in experimental porcine sepsis fulfill the RIFLE criteria for acute kidney injury? A systematic review
Source: Front Physiol. 2026 Aug 19;17:1810924. doi: 10.3389/fphys.2026.1810924 (PMC13533698; doi:10.3389/fphys.2026.1810924)
Supplement: Supplementary file 2 [file DataSheet2.pdf]

Supplemental Methods

Screening, study selection and eligibility criteria

All references were exported to EndNote (version 9.7.4; Thomson Reuters, Toronto, ON, Canada), where duplicates were removed. Screening was then performed in two stages (title/abstract, full text) in Microsoft Excel using an in-house developed standardized template. Initially, 15 abstracts were sampled from the search results and screened by all screeners (KJ, VIM, HJB, ABK, LMY) independently. Evaluation and discussion thereafter were applied to enhance consistency and reproducibility for the remaining screening process. Thereafter, titles and abstracts from all retrieved references were independently screened by screeners working in pairs. The screeners were blinded to each other's results. Records were at this stage selected for inclusion if all the inclusion criteria were or could be fulfilled, and none of the exclusion criteria were fulfilled. The inclusion and exclusion criteria are provided in Table 1. All other reports were excluded. Disagreements were resolved by consensus between the two screeners, unresolved disagreements following this stage resulted in inclusion to full-text review.

Two pair of authors (VIM/HJB and ABK/LMY) who were blinded to each other's results subsequently evaluated the full-text articles of the remaining relevant publications for eligibility. Articles were at this stage included in the review if all the inclusion criteria were fulfilled, and none of the exclusion criteria were fulfilled (Table 1). Conflicts were resolved by consensus and discussion. 897 records were screened, of which 59 were included in the final review. 309 records were identified from the reference lists of the included records. Two authors (VIM/LMY) who were blinded to each other's results performed screening of titles, abstracts, and full-text

articles using the same methodology as described above. An additional 5 records were identified and included in the systematic review, which ultimately contained a total of 64 records.

Screening procedure justification

To balance scientific rigor with practical feasibility, we designed a screening process that included multiple safeguards against bias and inconsistency. Dual independent screening was applied in both stages (title/abstract and full-text) to reduce selection bias and enhance reliability. Screening was conducted in Excel, which offered full transparency and control over the process, including the ability to log detailed justifications. This facilitated quality assurance and auditability throughout the review. All authors who screened followed predefined inclusion and exclusion criteria (table 1), which had been developed iteratively through plenary discussion. This ensured consistency and reproducibility across decisions. Overall, the screening approach was designed to minimize bias while remaining manageable within the project's scope and resources.

Data extraction and synthesis

Data extraction was performed by one of the authors (VIM or HJB) together with a second author (LMY). Consensus was sought with a third author (KJ) when needed. Only the data from the included articles were collected, study investigators were not contacted for obtaining or confirming data. From each study, the following information was extracted: first author, year of publication, country, sample size, animal characteristics, experimental length, intravenous fluids, anaesthesia, sepsis induction method, markers of kidney function used, creatinine/urine output/GFR data to be

used for RIFLE assessment, claim of renal dysfunction and if so whether the claim was supported by use of a defined criterion or classification. “Claim of renal dysfunction” was defined as every statement that clearly denoted a reduced function of the kidneys in any form in an included group (e.g. AKI, ARF, kidney failure, tubular injury, impaired/compromised renal function, renal dysfunction). All results that were compatible with each outcome domain in each study were sought. The selected studies were classified according to whether RIFLE criteria were fulfilled or not. Studies which fulfilled the RIFLE criteria were subsequently classified according to the risk, injury and failure criteria. This was performed by applying either the mean or the median data from the group in question, depending on what was available.

Due to the heterogeneity and imprecisions in the reported data the following steps were applied to detail extraction, interpretation and flesh out inclusion criteria for data extraction: For studies that only reported data on the individual animals, we calculated a mean of the data in question (e.g. urine output), which was applied for analysis in the review. If the renal function parameter was only provided as a graph, the graph was interpreted against the RIFLE criteria to the best of our abilities. Such studies were only excluded if data extractors in pairs deemed the graphs too unprecise to make a clear decision on RIFLE grade, or as to whether the group met at least the RIFLE Risk criteria. Studies in which urinary output was the only reported RIFLE parameter were excluded if the urine collection period from baseline was <6 hours, as the RIFLE criteria could not be assessed. If urine output was reported in mL/h, we calculated mL/kg/h by applying the weight of the lightest animal reported. Studies reporting creatinine using normalisations that changes over time (e.g. creatinine normalised for plasma-protein) were excluded as the fulfilment of RIFLE

criteria could be secondary to changes in the normalised parameter and not the renal functional parameter itself. Studies in which sepsis was combined with another primary inflammatory model (e.g. major burns) were deemed to not aim at creating sepsis and was excluded from this review. Missing data were recorded as missing and not included in statistical analysis. The record was considered a duplicate if the group fulfilling the inclusion criteria already had been included for the review through a separate record.

Quality assessment

Several risks of bias tools exist for use in systematic reviews on animals. After reviewing alternatives such as the GRADE approach, the animal study specific SciRAP and SYRCLE and the observational study specific ROBINS-I or Newcastle-Ottawa scale, we did not identify tools appropriate for use with our study design. Unlike what is presumed in the mentioned risk of bias tools, this systematic review does not investigate differences between groups within each included article. Instead, it focuses on data reported on renal function in a single group with untreated sepsis. Therefore, it is not appropriate to exclude articles on grounds used in the available tools (e.g. lack of sample size estimation, group allocation), making the tools obsolete for the purpose of the current review. Furthermore, another goal for this review was to document whether renal dysfunction was claimed by the authors and which specific criterion (e.g. RIFLE criteria) that eventually were applied. Exclusion of papers deemed of inferior quality would thus defeat the purpose of the review. Therefore, no formal risk of bias or reporting quality assessment was conducted. Peer review was a criterion for inclusion, which provided a certain degree of quality control to mitigate the omission of quality assessment.

Statistics

Statistical analyses were performed using STATA (MP18.0 for Mac, StataCorp LLC). All analyses were conducted at a study (group) level. For each included study, a binary outcome was defined indicating whether at least one RIFLE stage was fulfilled. Descriptive statistics were used to summarize the distribution of RIFLE outcomes and study characteristics. Associations between reporting of individual renal parameters and fulfilment of RIFLE criteria were explored using contingency tables with Fisher's exact test. Study-level logistic regression was used as a complementary approach to estimate odds ratio and confidence intervals for these associations. Univariable logistic regression was used to assess the association between claimed renal dysfunction and fulfilment of at least RIFLE Risk. Effect estimate was presented as odds ratio (OR). P-values < 0.05 were considered statistically significant.

Meta-analysis

A meta-analysis of study-level proportions was performed to estimate the pooled proportion of studies fulfilling at least RIFLE Risk [1]. Because a substantial number of studies reported zero events (i.e. no fulfilment of RIFLE), proportions were analysed on the logit scale to ensure valid variance estimation and confidence intervals. The number of animals per study was not incorporated as a weighting factor, as the outcome was defined at the study level and within-study variability could not be quantified. Study size was therefore explored only in sensitivity analyses.

Random-effects models were fitted using restricted maximum likelihood. Pooled estimates were back-transformed to the proportion scale for interpretation. Subgroup meta-analyses were conducted according to sepsis induction method. Differences between subgroups were formally assessed using tests of between-group heterogeneity. All included studies applied either Escherichia coli (E. coli), Pseudomonas Aeruginosa (P. Aeruginosa), lipopolysaccharide (LPS) or fecal contamination, except one study that applied group A streptococci intravenously. This latter study was not included in the subgroup meta-analyses as it was deemed to not provide any information. Sensitivity analyses were performed to explore whether the number of animals in the included studies had any impact on the probability of fulfilling the RIFLE criteria. In addition, the same sensitivity analyses were repeated using the square root-transformed study size to account for outliers in number of animals.

Supplemental Results

The most common used animal strains among RIFLE positive studies were "domestic pigs", followed by "Yorkshire pigs". Number of animals included in each study varied considerably. The smallest studies included only four pigs, while 20 pigs were included in the largest cohort. Six of the 30 studies which fulfilled the RIFLE criteria were performed before RIFLE was published in 2004[2].

Studies which fulfilled the RIFLE Risk criteria

16 studies fulfilled the RIFLE Risk criteria. All studies were published between 1989 and 2024. 11 studies were performed in domestic pigs weighing 20-60 kg, while four studies[3-6] were performed in piglets weighing 2.5-3.5 kg. Renal function was reported as creatinine clearance in three studies[5-7], while creatinine and/or urine output were reported in the remaining studies. A final study applied iothalamate[4]. However, interpretation of urine output data was quite challenging in several of the studies due to imprecise reporting.

Main objectives for studies which fulfilled the RIFLE risk criteria varied. In the study by Chin et al[4] the main objective was to study the effects of dopamine on renal function in neonatal E. coli sepsis. This was the only study in which GFR was monitored by a precise exogenous substance (iothalamate). Fanous et al[3] aimed to validate a novel transdermal GFR monitoring device in neonatal pigs. Sepsis was induced by cecal puncture and animals observed for 12 hours. Research objectives in the remaining studies (adult pigs) focused on the effects and mechanisms of plasma exchange, immunoneutralization, free radical scavengers, carbon monoxide,

oxygen fraction, hypertonic sodium lactate, mesenchymal stem cells, contrast-enhanced ultrasound imaging, renal progenitor cells, and high-dose vasopressors.

Studies which fulfilled the RIFLE Injury criteria

Nine studies fulfilled the RIFLE Injury criteria. Pigs weighing 26-74 kg were included. One study included piglets at 1.9-2.5 kg[8]. All studies were published between 1998 and 2024. An exogenous marker for GFR monitoring was applied in two studies[8,9]. Three studies reported creatinine clearance data, while three studies reported both creatinine and urine output data.

In the study by Martinez et al[10] RIFLE injury was fulfilled for the urine output criteria and the authors claimed “oliguric renal failure”. In the study by Lipcsey et al[11] creatinine clearance was >50% reduced 6 hrs after sepsis induction, which fulfilled the GFR criteria. However, AKI was not claimed by the authors. Fenhammar et al[12] studied pigs for 5 hrs after sepsis induction. Creatinine clearance was 115 mL/min at baseline and 50 mL/min after 5 hrs. The GFR criteria for RIFLE injury was thus fulfilled. AKI was claimed by the authors, but without any reference to any specific criterion. Sølling et al[9] induced sepsis in 10 pigs and observed the animals for 10 hrs. GFR was calculated with the use of ^{51}Cr-EDTA and showed >50% reduction from baseline. The authors claimed renal dysfunction without referring to any specific definition. Granfeldt et al[13] performed 8 experiments and observed the animals for 5 hours. Creatinine clearance at baseline was 120 mL/min decreasing to 58 mL/min after 300 minutes, which is a 52% reduction. The authors used the terms “impaired” and “drop in renal function” to describe renal dysfunction in this model. Stasi et al[14] studied six animals for 24 hours. Despite lack of baseline values, urine output was

<0.5 mL/kg/h from 0 to 24 hours. RIFLE injury criteria were thus fulfilled. The authors claimed “renal dysfunction”, but without any reference to any specific definition. Olney et al[15] studied four pigs for 350 minutes. Creatinine increased 131.5% from baseline. Acute kidney injury was claimed by the authors, but without any reference to the RIFLE criteria. Sugita et al[16] studied five animals for 4 hours. Creatinine increased from 0.77 mg/dL to 1.60, which represents an increase of 108%. Authors claimed AKI without any reference to a definition.

Studies which fulfilled the RIFLE Failure criteria

Five studies fulfilled the RIFLE Failure criteria. Studies were published 2013-2025 and included pigs weighing 18-60 kg. Three studies reported creatinine clearance data, while the remaining two studies reported creatinine and urine output data. Exogenous GFR markers were not applied in any of the studies.

Wang et al[17] studied six pigs for 24 hours. Plasma creatinine at baseline was 70.0 ± 3.9 mmol/L increasing to 211.0 ± 21.7 at 24 hrs. The authors claimed “organ damage” without reference to the RIFLE criteria. Vassal et al[18] studied six pigs weighing 20-25 kg. Intravenous infusion of live P. aeruginosa induced sepsis and the animals were studied for 160 minutes. Creatinine clearance at baseline was 63.6 decreasing to 12.0 after 160 minutes. Sepsis induced AKI was claimed by the authors without any reference to the RIFLE criteria. Duburcq et al[19] studied five pigs weighing 18-24 kg. Sepsis was induced by intravenous infusion of E. coli lipopolysaccharide, and the animals were studied for 5 hours. Creatinine clearance at baseline was 95 mL/min and decreased to 2 mL/min after 5 hours. The description of renal impairment by the authors was without any reference to a specific criterion. Hong et al[20] studied 12 pigs

weighing 55-60 kg. Sepsis was induced by infusion of live Pseudomonas aeruginosa, and the animals were studied for 24 hours. Creatinine at baseline was 84.2 μmol/L and increased to 439.9 after 22 hours. RIFLE failure criteria fulfilled, but AKI or injury were not claimed by the authors. Von Seth et al[21] studied 6 animals weighing 22.3-29.3 kg. Sepsis was induced by an intravenous infusion of E. coli LPS. Creatinine clearance at baseline was 71μmol/L which decreased to 35 μmol/L after 6 hours. The authors claimed "organ dysfunction".

Hemodynamics, oxygen transport, fluid resuscitation, fluid balance and anaesthesia

Hemodynamic data, oxygen transport variables, type of resuscitation fluids and total fluid replacement volumes varied enormously within and between RIFLE categories. Any formal statistical comparisons were not possible due to large variation in the way data were recorded and presented. Colloids (e.g. hydroxyethyl starch) were used quite frequently in several of the experimental models. This is interesting as hypervolemia and use of colloids are both associated with AKI[22]. Anesthesia protocols varied considerably among included studies, which may provide unwanted impact on renal function as the type of anaesthesia may be relevant for development of porcine AKI models[23].

References

1. Barker TH, Migliavaca CB, Stein C, et al. Conducting proportional meta-analysis in different types of systematic reviews: a guide for synthesisers of evidence. *BMC Medical Research Methodology.* 2021;21(1):189.
2. Bellomo R, Ronco C, Kellum JA, Mehta RL, Palevsky P, Acute Dialysis Quality Initiative w. Acute renal failure - definition, outcome measures, animal models, fluid therapy and information technology needs: the Second International Consensus Conference of the Acute Dialysis Quality Initiative (ADQI) Group. *Crit Care.* 2004;8(4):R204-212.
3. Fanous MS, Afolabi JM, Michael OS, Falayi OO, Iwhiwhu SA, Adebiyi A. Transdermal Measurement of Glomerular Filtration Rate in Mechanically Ventilated Piglets. *Journal of visualized experiments : JoVE.* 2022(pagination).
4. Chin A, Nguyen O'conner L, Radhakrishnan J, Fornell L, John E. Endotoxemia and the effects of dopamine on renal functions of neonatal piglets. *Biology of the Neonate.* 2002;81(3):196-202.
5. Furtado N, Beier UH, Gorla SR, et al. The effect of indomethacin on systemic and renal hemodynamics in neonatal piglets during experimental endotoxemia. *Pediatric Surgery International.* 2008;24(8):907-911.
6. John E, Pais P, Furtado N, et al. Early effects of lipopolysaccharide on cytokine release, hemodynamic and renal function in newborn piglets. *Neonatology.* 2008;93(2):106-112.
7. Barth E, Bassi G, Maybauer DM, et al. Effects of ventilation with 100% oxygen during early hyperdynamic porcine fecal peritonitis. *Critical Care Medicine.* 2008;36(2):495-503.
8. Lievano G, Nguyen L, Radhakrishnan J, Fornell L, Joshi A, John EG. Significance of fractional excretion of sodium and endothelin levels in the early diagnosis of renal failure in septic neonatal piglets. *Journal of Pediatric Surgery.* 1998;33(10):1480-1482.
9. Solling C, Christensen AT, Nygaard U, et al. Erythropoietin does not attenuate renal dysfunction or inflammation in a porcine model of endotoxemia. *Acta Anaesthesiologica Scandinavica.* 2011;55(4):411-421.
10. Martinez JM, Wagner KE, Snider RH, et al. Late immunoneutralization of procalcitonin arrest the progression of lethal porcine sepsis. *Surgical Infections.* 2001;2(3):193-203.
11. Lipcsey M, Larsson A, Olovsson M, Sjölin J, Eriksson MB. Early endotoxin-mediated haemostatic and inflammatory responses in the clopidogrel-treated pig. *Platelets.* 2005;16(7):408-414.
12. Fenhammar J, Andersson A, Forestier J, et al. Endothelin receptor a Antagonism attenuates renal medullary blood flow impairment in Endotoxemic pigs. *PLoS ONE.* 2011;6(7) (no pagination).
13. Granfeldt A, Letson HL, Dobson GP, Shi W, Vinten-Johansen J, Tonnesen E. Adenosine, lidocaine and Mg2+ improves cardiac and pulmonary function, induces reversible hypotension and exerts anti-inflammatory effects in an endotoxemic porcine model. *Critical Care (London, England).* 2014;18(6):682.
14. Stasi A, Franzin R, Fiorentino M, et al. Recombinant High-Density Lipoproteinmodulates Inflammatory Response and Renal Dysfunction in a

Swinemodel of Sepsis-Induced Acute Kidney Injury. *Nephrology Dialysis Transplantation*. 2023;38(Supplement 1):i90.

15. Olney KC, de Avila C, Todd KT, et al. Commonly disrupted pathways in brain and kidney in a pig model of systemic endotoxemia. *Journal of Neuroinflammation*. 2024;21(1) (no pagination).
16. Sugita C, Itami T, Miyasho T, et al. The anti-inflammatory effects of Fuzapladib in an endotoxemic porcine model. *Journal of Veterinary Medical Science*. 2024;86(11):1145-1155.
17. Wang W, Zhao F, Fang Y, et al. Glycyrrhizin protects against porcine endotoxemia through modulation of systemic inflammatory response. *Critical Care*. 2013;17(2).
18. Vassal O, Bonnet JM, Barthelemy A, et al. Renal haemodynamic response to amino acids infusion in an experimental porcine model of septic shock. *Acta Anaesthesiologica Scandinavica*. 2015;59(5):598-608.
19. Duburcq T, Durand A, Tournoys A, et al. Sodium lactate improves renal microvascular thrombosis compared to sodium bicarbonate and 0.9% NaCl in a porcine model of endotoxic shock: an experimental randomized open label controlled study. *Annals of Intensive Care*. 2018;8(1) (no pagination).
20. Hong SL, Dumargne H, Hahn RG, et al. Volume kinetics in a translational porcine model of stabilized sepsis with fluid accumulation. *Critical Care*. 2025;29(1) (no pagination).
21. von Seth M, Sjolin J, Larsson A, Eriksson M, Hillered L, Lipcsey M. Effects of tigecycline and doxycycline on inflammation and hemodynamics in porcine endotoxemia: a prospective, randomized, and placebo-controlled trial. *Shock*. 2015;43(6):604-611.
22. Perner A, Haase N, Guttormsen AB, et al. Hydroxyethyl starch 130/0.42 versus Ringer's acetate in severe sepsis. *N Engl J Med*. 2012;367(2):124-134.
23. Guilpin A, Magnin M, Aigle A, et al. Impact of different anesthetic protocols during anesthesia for the establishment of a porcine model of acute kidney injury. *Animal Model Exp Med*. 2025;8(8):1493-1502.
